# Optimization of Organic Photodetector Performance Using SCAPS 1D Simulation: Enhanced Quantum Efficiency and Responsivity for UV Detection

**DOI:** 10.3390/nano16050324

**Published:** 2026-03-04

**Authors:** Ahmet Sait Alali, Fedai Inanir

**Affiliations:** Department of Physics, Yıldız Technical University, Istanbul 34220, Turkey; saitnuclear@gmail.com

**Keywords:** organic photodetector, SCAPS 1D simulation, quantum efficiency, responsivity, UV detection, PTB7, Spiro-OMeTAD, device optimization

## Abstract

This study presents a SCAPS-1D-based numerical optimization of an organic ultraviolet (UV) photodetector employing an FTO/PTB7/Spiro-OMeTAD/Au device architecture. The novelty of this work lies in a simulation-guided, UV-specific optimization strategy that combines thickness engineering, controlled doping, and contact work-function tuning to achieve intrinsic spectral selectivity without external optical filters. We systematically optimize material and device parameters, including active layer thicknesses, donor and acceptor densities, and the metal electrode work function, to enhance responsivity, detectivity, and spectral performance. Simulations identify optimal thicknesses of 1200 nm for PTB7 and 1000 nm for Spiro-OMeTAD, with donor concentrations of 1 × 10^20^ cm^−3^ and 1 × 10^18^ cm^−3^, respectively. A comparative contact analysis demonstrates that replacing aluminum with gold (Au) forms a near-ohmic back contact, leading to improved hole extraction and suppressed dark current due to favorable energy-level alignment. The optimized device achieves a peak external quantum efficiency of approximately 80% in the 300–400 nm ultraviolet range, with a responsivity up to 0.4 A/W. The UV selectivity originates from the absorption characteristics of PTB7 combined with suppressed long-wavelength charge collection, resulting in a negligible response in the visible–near-infrared region. These results confirm the device’s strong potential for high-sensitivity, solar-blind UV photodetection. By integrating practical material selection with physically consistent SCAPS-1D optoelectronic modeling, this work provides a robust design framework to guide the development of next-generation organic UV photodetectors for environmental sensing, biomedical diagnostics, and wearable optoelectronics.

## 1. Introduction

Organic photodetectors (OPDs) have emerged as a promising alternative to conventional inorganic photodetectors due to their mechanical flexibility, lightweight nature, low-temperature solution processability, and tunable spectral response ranging from ultraviolet (UV) to near-infrared (NIR) wavelengths [1]. These advantages make OPDs attractive for applications including imaging systems, wearable health monitoring, environmental sensing, flame detection, and UV-specific detection technologies [2]. Compared with silicon and III–V semiconductor photodetectors, which require costly fabrication and rigid substrates, organic semiconductors enable large-area, flexible, and conformable photodetection platforms [3].

The performance of photodetectors is commonly evaluated using key figures of merit such as external quantum efficiency (EQE), responsivity (R), specific detectivity (D*), dark current, and response time. EQE quantifies the fraction of incident photons converted into collected charge carriers, with values exceeding 100% reported in photomultiplication-type OPDs [4]. Responsivity (A W^−1^) describes the electrical output per unit optical input power [5], while specific detectivity (Jones) incorporates both responsivity and noise current to assess detector sensitivity [6]. Dark current is a dominant noise source that limits detectivity, particularly in low-light and UV detection applications [7]. Recent advances have demonstrated visible-blind UV OPDs with detectivity exceeding 10^12^ Jones and photomultiplication-type devices achieving extremely high EQEs at UV wavelengths, highlighting the rapid progress in this field [8,9].

UV photodetection represents a particularly important application area for OPDs due to increasing demands in flame monitoring, environmental surveillance, secure communication, and biomedical diagnostics [10]. Solar-blind and visible-blind UV detectors are especially desirable because they suppress background radiation from visible and infrared light, thereby improving signal-to-noise ratios [11]. Recent studies have reported flexible and high-resolution UV imaging OPDs suitable for wearable and portable systems [12]. Achieving intrinsic UV selectivity without external optical filters remains a key challenge and requires careful optimization of material absorption, device architecture, and carrier transport mechanisms [13].

The choice of active layer materials critically determines OPD performance. PTB7 and its derivatives are widely studied donor polymers due to their favorable optoelectronic properties, including a narrow bandgap (~1.81 eV), high absorption coefficient, good charge transport characteristics, and excellent film-forming ability [14,15]. PTB7-based OPDs have demonstrated high responsivity and low dark current, while ternary blend strategies have enabled broadband detection extending from UV to NIR wavelengths [16,17]. Despite these advances, the systematic optimization of PTB7-based planar heterojunction architectures for UV-selective photodetection remains relatively unexplored, particularly from a physics-based simulation perspective.

Device architecture and interfacial engineering play equally critical roles in determining OPD performance. Hole transport layers (HTLs) enhance selective hole extraction while blocking electrons, thereby reducing recombination and suppressing dark current. Spiro-OMeTAD is a widely used HTL due to its suitable energy-level alignment (HOMO ≈ 5.0 eV), wide bandgap (~3.2 eV), high optical transparency, and good thermal stability [18,19]. The doping level and thickness of the HTL strongly influence band bending, internal electric field distribution, and contact resistance, making their optimization essential for UV-sensitive OPDs.

Computational modeling has become an indispensable tool for accelerating OPD optimization. The Solar Cell Capacitance Simulator in one dimension (SCAPS-1D) is widely employed to analyze thin-film optoelectronic devices by solving Poisson’s and carrier continuity equations under steady-state and illuminated conditions [20,21]. SCAPS-1D enables systematic evaluation of layer thicknesses, doping profiles, defect states, interface recombination, and electrode work functions. While SCAPS-1D has been extensively applied to solar cells and visible-range OPDs, its use for UV-selective OPDs with detailed contact and doping engineering remains limited [22].

Despite substantial progress, challenges persist in OPD development, particularly the trade-off between high gain and dark current suppression, as well as achieving stable, low-bias UV operation [23,24]. Moreover, the combined effects of donor/acceptor doping, interface properties, and metal electrode work function on internal electric field formation and spectral selectivity have not been comprehensively investigated for PTB7-based OPDs [25,26].

In this work, we present a comprehensive SCAPS-1D simulation study to optimize an organic UV photodetector based on an FTO/PTB7/Spiro-OMeTAD architecture, with a systematic comparison between Al and Au back contacts. By investigating the effects of active layer thickness, HTL thickness, doping concentrations, interface behavior, and electrode work function, we identify device configurations that maximize EQE, responsivity, and UV selectivity while suppressing dark current. The results provide physics-based design guidelines for high-performance, intrinsically UV-selective organic photodetectors and support future experimental implementation.

## 2. Methodology

### 2.1. Simulation Software and Computational Approach

The numerical simulations presented in this study were performed using the Solar Cell Capacitance Simulator in one dimension (SCAPS-1D), version 3.3.07, developed at the University of Gent, Belgium [27]. SCAPS-1D is a widely adopted simulation platform that solves the fundamental semiconductor equations—Poisson’s equation and the electron and hole continuity equations—under steady-state and transient conditions to predict the optoelectronic behavior of multilayer thin-film devices [28]. The software has been extensively validated for simulating organic photovoltaic devices and photodetectors, enabling systematic investigation of how material properties, layer thicknesses, doping concentrations, defect densities, interface characteristics, and electrode work functions influence device performance [29].

The core equations solved by SCAPS-1D include:

Poisson’s equation:

(1) $$d2\psi dx2=-q\varepsilon_{0}\varepsilon_{r}\left(p-n+{ND}^{+}+{NA}^{-}+\rho trap\right)$$

where *ψ* is the electrostatic potential, *q* is the elementary charge, *ε*_0_ is the vacuum permittivity, *ε*_r_ is the relative dielectric constant, *p* and *n* are the hole and electron concentrations, ${ND}^{+}$ and ${NA}^{-}$ are the ionized donor and acceptor concentrations, and ρtrap represents the charge density associated with trap states.

(2) dJndx=q(G−R+∂n∂t)

(3) dJpdx=−q(G−R+∂p∂t)

where *Jn* and *Jp* are the electron and hole current densities, *G* is the generation rate, and *R* is the recombination rate.

The simulation assumes one-dimensional carrier transport perpendicular to the device layers, appropriate for planar multilayer structures. Optical generation profiles are calculated using the transfer matrix method, accounting for wavelength-dependent absorption coefficients and refractive indices of each layer [30]. Recombination mechanisms considered include direct band-to-band recombination, Shockley–Read–Hall (SRH) trap-assisted recombination, and interface recombination at layer boundaries [31].

### 2.2. Device Architecture and Layer Configuration

**Figure 1:**
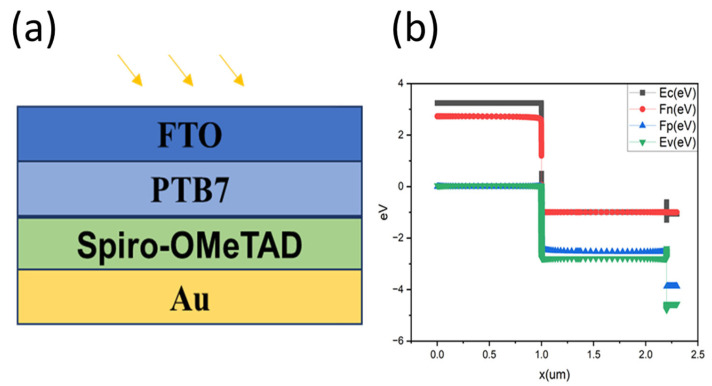
(**a**) Schematic structure of the device and (**b**) energy band diagram.

The organic photodetector structure investigated in this study is based on a planar FTO/PTB7/Spiro-OMeTAD architecture, with the metal back contact varied between aluminum (Al) and gold (Au) to evaluate the effect of electrode work function on device performance. Al is used as a reference low-work-function electrode, while Au represents the optimized back contact, as illustrated in Figure 1.

Fluorine-doped tin oxide (FTO) serves as the transparent front electrode and electron-collecting contact. FTO provides high optical transparency in the UV–visible–NIR range (transmittance > 80% for λ > 400 nm), good electrical conductivity (sheet resistance ~10–15 Ω/sq), chemical stability, and an appropriate work function (~4.4 eV) for electron extraction [32]. Its wide bandgap and high electron affinity facilitate efficient electron collection while blocking hole transport toward the cathode.

PTB7 functions as the photoactive donor layer. It was selected due to its narrow bandgap (1.81 eV), strong absorption coefficient (α > 10^5^ cm^−1^), favorable charge transport properties, and demonstrated performance in organic optoelectronic devices [33]. The donor–acceptor molecular structure and extended π-conjugation of PTB7 enable efficient exciton generation and dissociation upon photon absorption.

Spiro-OMeTAD acts as the hole transport layer (HTL). It exhibits a wide bandgap (~3.2 eV), a HOMO level of approximately 5.0 eV well aligned with PTB7, high hole mobility (~2 × 10^−4^ cm^2^ V^−1^ s^−1^), excellent optical transparency in the UV–visible region, and strong electron-blocking capability [34]. These properties make Spiro-OMeTAD particularly suitable for UV photodetector applications.

In the optimized configuration, gold (Au) is employed as the back-contact anode. Au possesses a high work function (~5.1–5.4 eV), which aligns favorably with the HOMO level of Spiro-OMeTAD, enabling near-ohmic hole extraction and suppressing interfacial recombination losses. This energetic alignment significantly reduces contact-induced dark current and improves charge collection efficiency. In addition, the high optical reflectivity of Au enhances light management by reflecting unabsorbed photons back into the active layer, effectively increasing the optical path length [35].

Figure 1b presents the energy band diagram corresponding to the optimized FTO/PTB7/Spiro-OMeTAD/Au device. Upon UV illumination, excitons are generated in the PTB7 layer and dissociate at the PTB7/Spiro-OMeTAD interface. The built-in electric field, established primarily by the work-function difference between the FTO front contact and the Au back contact, drives electrons toward the FTO electrode and holes toward the Au anode through the Spiro-OMeTAD layer.

### 2.3. Material Parameters and Physical Properties

Table 1 summarizes the comprehensive set of physical and electronic parameters utilized in the SCAPS-1D simulations. These parameters were extracted from the experimental literature and theoretical studies to ensure realistic modeling of device behavior.

The electron affinity values determine the conduction band offsets at the heterojunction interfaces, while the bandgap values define the relative valence band positions and thus govern carrier injection and extraction barriers. The effective density of state parameters control carrier statistics and the occupancy of band-edge states, directly influencing recombination and transport behavior. Carrier mobilities, obtained from space-charge-limited current (SCLC) measurements and field-effect transistor characterizations reported in the literature, govern charge transport efficiency and transit time within each layer [39]. The thermal velocities represent the average carrier velocities under thermal equilibrium and affect capture rates and recombination kinetics in the presence of defect states.

Defect states within the bandgap act as recombination centers and trapping sites, significantly impacting charge collection efficiency and dark current. To ensure physical consistency while avoiding overparameterization, a single effective mid-gap defect level with a neutral charge state was assumed for each layer, following commonly adopted SCAPS-1D modeling practices. The defect density was set to 1.0 × 10^15^ cm^−3^, with electron and hole capture cross-sections of 1.0 × 10^−15^ cm^2^, and an energetic distribution width of 0.1 eV [40]. These values represent typical defect characteristics of solution-processed organic semiconductors and allow meaningful comparison of relative device trends rather than absolute performance prediction.

### 2.4. Interface Characteristics

Interface recombination at the FTO/PTB7 and PTB7/Spiro-OMeTAD heterojunctions plays a critical role in determining charge collection efficiency, internal electric field distribution, and dark current behavior in organic photodetectors. Carrier recombination at these interfaces is particularly important in UV photodetectors, where photogenerated carriers are often created close to interfaces due to short absorption depths. Interface defect states were modeled in SCAPS-1D as continuous distributions within the bandgap, characterized by the interface defect density (NitN_{it}Nit), electron and hole capture cross-sections, and energetic positions [41].

For the baseline simulations, an identical interface defect density of 1.0 × 10^11^ cm^−2^ was assumed at both the FTO/PTB7 and PTB7/Spiro-OMeTAD interfaces, with electron and hole capture cross-sections of 1.0 × 10^−15^ cm^2^. These values were selected to represent moderately well-formed organic heterojunctions and to avoid artificially suppressing recombination, thereby ensuring physically realistic device operation. The interface recombination velocity, which quantifies the rate of carrier annihilation at the interface, was set to 1.0 × 10^5^ cm/s, consistent with commonly reported values for organic semiconductor interfaces.

To isolate the effects of layer thickness, doping concentration, and electrode work function, interface parameters were kept constant throughout all parametric studies. This approach allows performance variations to be directly attributed to the investigated design parameters rather than to uncontrolled interface changes. The influence of interface recombination on device behavior is therefore implicitly captured through its impact on dark current, EQE, and responsivity, particularly under low-bias UV illumination conditions.

### 2.5. Optical Properties and Generation Profile

The wavelength-dependent complex refractive indices ($n^{+}i\kappa$, where n is the real part and *κ* is the extinction coefficient) for FTO, PTB7, and Spiro-OMeTAD were incorporated into the simulation to accurately model optical absorption, reflection, and interference effects within the multilayer device structure [42]. The absorption coefficient *α*(*λ*) is related to the extinction coefficient through

(4) $$\alpha =\frac{4\pi \kappa }{\lambda }$$

Spectral simulations were performed over the wavelength range of 300–800 nm, covering the ultraviolet and visible regions relevant to photodetector operation. This range enables evaluation of UV selectivity as well as suppression of long-wavelength response.

The photogeneration rate *G*(*x*,*λ*) as a function of position x and wavelength *λ* was calculated inherently within SCAPS-1D using the transfer-matrix formalism, which accounts for multiple reflections and optical interference in the multilayer stack [43]:

(5) $$G(x,\lambda )\;=\;[\;2\;\alpha (\lambda )\;{\left|E(x,\lambda )\right|}^{2}]/(\hbar \omega )$$

where *E*(*x*,*λ*) is the spatially resolved electric field intensity distribution, ħ*ω* is the photon energy, and the prefactor converts absorbed optical energy into the corresponding charge-carrier generation rate. As a result, the carrier generation profile is self-consistently coupled to layer thickness, optical constants, and wavelength, without requiring any external optical simulations. This is particularly important for UV photodetectors, where short absorption depths lead to carrier generation close to interfaces and strongly influence recombination and collection efficiency.

### 2.6. Simulation Conditions and Parametric Studies

All simulations were conducted under standard operating conditions using AM1.5G illumination (100 mW cm^−2^), a temperature of 300 K, and a voltage sweep from −1 V to +1 V with a step size of 0.01 V. Although UV photodetectors often operate under narrow-band illumination, the AM1.5G spectrum was employed to enable consistent evaluation of spectral selectivity and broadband suppression within the SCAPS-1D framework. The convergence criteria for the iterative solution of the coupled Poisson and continuity equations were set to 10^−6^ for relative errors in electrostatic potential and carrier concentrations.

To systematically optimize device performance, parametric studies were carried out by independently varying key structural and material parameters while keeping all other parameters fixed:

Spiro-OMeTAD layer thickness optimization:

The hole transport layer (HTL) thickness was varied from 50 nm to 1000 nm in steps of 50 nm. This study evaluates the trade-off between efficient hole extraction and increased series resistance and recombination associated with excessive transport length.

PTB7 layer thickness optimization:

The PTB7 active layer thickness was swept from 50 nm to 1200 nm in steps of 50 nm, enabling identification of the optimal balance between enhanced optical absorption (favoring thicker layers) and efficient charge collection (favoring thinner layers to minimize recombination losses).

PTB7 donor density optimization:

The shallow donor concentration in the PTB7 layer was varied from 1.0 × 10^14^ to 1.0 × 10^21^ cm^−3^, allowing evaluation of how doping-induced band bending and internal electric field strength influence carrier separation, recombination, and photocurrent generation.

Interface parameters and defect densities were kept constant throughout all parametric studies to ensure that observed performance variations arise solely from the investigated design parameters.

For each parametric variation, the following performance metrics were extracted and analyzed:

Current density–voltage (J–V) characteristics under dark and illuminated conditions;

Short-circuit current density (Jsc), representing maximum photocurrent generation;

External quantum efficiency (*EQE*) as a function of wavelength;

Spectral responsivity *R*(*λ*), calculated from *EQE* as

(6) R(λ)=(q/hc)×λ×EQE(λ)

where

*q* is the elementary charge, *h* is Planck’s constant, and *c* is the speed of light;
Peak responsivity and corresponding wavelength;
Dark current density at zero bias and reverse bias (−1 V).

### 2.7. Performance Metrics and Analysis

The key figures of merit used to evaluate photodetector performance were calculated directly from the SCAPS-1D simulation outputs.

The external quantum efficiency (*EQE*) is defined as

(7) $$EQE\left(\lambda \right)=\frac{Jph\left(\lambda \right)}{q\Phi \left(\lambda \right)}\times 100\%$$

where $Jph\left(\lambda \right)$ is the wavelength-dependent photocurrent density, *q* is the elementary charge, and $\Phi \left(\lambda \right)$ is the incident photon flux.

The spectral responsivity *R*(*λ*) is given by

(8) $$R\left(\lambda \right)=Jph\left(\lambda \right)Pin\left(\lambda \right)$$

where $Pin\left(\lambda \right)$ is the incident optical power density.

The specific detectivity D*, which quantifies the signal-to-noise ratio normalized to detector area and bandwidth, is expressed as

(9) $$D^{*}=RA\cdot \Delta fin\;$$

where *A* is the active device area, Δ*f* is the electrical bandwidth, and in is the noise current. Assuming shot-noise-limited operation, the noise current is approximated as

(10) in-2qJdarkAΔf  

yielding

(11) $$D^{*}=R2qJdark$$

The primary optimization objective was to maximize EQE and responsivity in the ultraviolet region (300–400 nm) while minimizing dark current density, thereby achieving high specific detectivity suitable for sensitive UV photodetection. This metrics-based evaluation provides quantitative design guidelines for experimental fabrication and identifies the parameters that most strongly govern UV selectivity, charge collection efficiency, and noise suppression.

## 3. Results and Discussion

### 3.1. Thickness of the Spiro-OmeTad Layer

The thickness of the hole transport layer (HTL) plays a crucial role in determining the efficiency, sensitivity, and dark current behavior of organic photodetectors (OPDs). Optimizing the relative thickness of the photoactive layer and the HTL significantly influences charge extraction efficiency, internal electric field distribution, and recombination dynamics [44]. An excessively thin HTL may lead to incomplete coverage and increased interfacial recombination, whereas an overly thick HTL can introduce excessive carrier transport length and series resistance, thereby degrading device performance.

Previous studies have demonstrated that a careful control of transport layer thickness can effectively suppress dark current and enhance photoresponse. For example, thickness optimization in OPDs employing nonfused ring electron acceptors has been shown to reduce dark current and improve photoelectric characteristics [45]. Similarly, optimizing interfacial layer thickness in bulk heterojunction photodetectors has resulted in low dark current density, high specific detectivity, and fast response times [46]. In planar heterojunction devices, matching the transport layer thickness to the effective carrier diffusion length is particularly important to minimize bulk recombination and maximize charge collection efficiency, leading to significantly improved detectivity [47].

**Figure 2:**
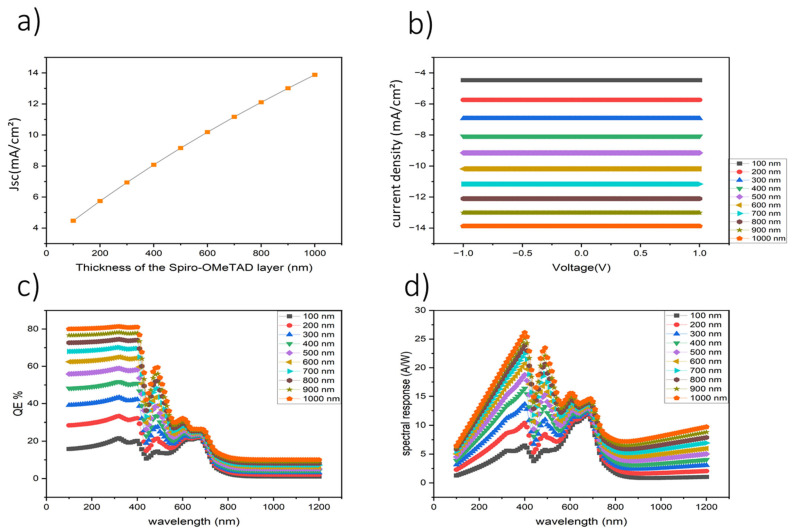
Influence of various Spiro-OmeTad thicknesses on device performance. (**a**) Thickness dependence of the current density. (**b**) Voltage–current density characteristics. (**c**) External quantum efficiency (EQE) as a function of wavelength. (**d**) Spectral response for different Spiro-OmeTad thicknesses.

Figure 2 illustrates the influence of Spiro-OMeTAD thickness on the optoelectronic performance of the simulated organic photodetector. Panel (a) shows that the photocurrent density increases with HTL thickness, reaching a maximum value of 13.87 mA cm^−2^ at a thickness of 1000 nm. This trend reflects improved hole extraction and reduced interfacial recombination as the Spiro-OMeTAD layer becomes sufficiently thick to form a continuous and energetically favorable transport pathway. However, thicknesses beyond this value do not yield further improvement due to increased carrier transport length and bulk recombination losses, which counteract the benefits of enhanced coverage.

Panel (b) presents the corresponding current density–voltage (J–V) characteristics, where thicker Spiro-OMeTAD layers up to 1000 nm exhibit higher and more stable photocurrent under applied bias, indicating efficient charge extraction and low series resistance within this thickness range. The absence of further current enhancement at larger thicknesses suggests the onset of transport limitations rather than optical absorption constraints, consistent with the low absorption coefficient of Spiro-OMeTAD.

Panel (c) shows the external quantum efficiency (EQE) spectra as a function of wavelength. The device with a 1000 nm Spiro-OMeTAD layer exhibits the highest EQE, reaching approximately 80% in the UV–visible region, confirming optimal charge collection efficiency at this thickness. Panel (d) presents the corresponding spectral responsivity, which follows the same trend as EQE and further supports the identification of 1000 nm as the optimal HTL thickness.

### 3.2. Optimization of the Thickness of the PTB7 Layer

**Figure 3:**
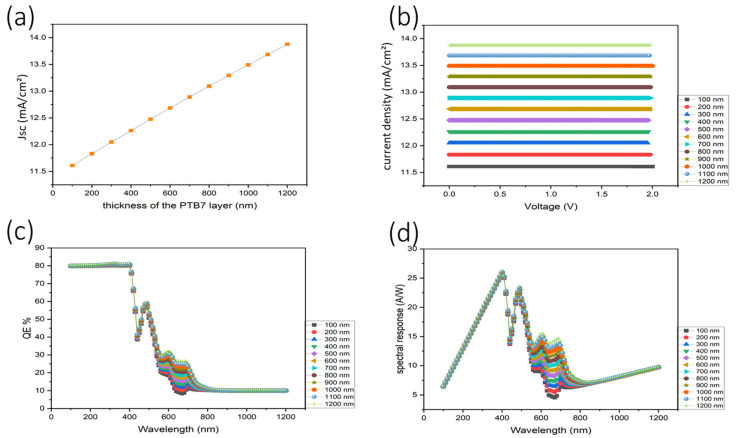
Impact of various PTB7 thicknesses on device performance. (**a**) Thickness dependence of the current density. (**b**) Voltage–current density characteristics. (**c**) External quantum efficiency (EQE) as a function of wavelength. (**d**) Spectral response for different PTB7 thicknesses.

Figure 3 presents a comprehensive evaluation of the impact of PTB7 layer thickness on the performance metrics of the simulated organic photodetector (OPD). Panel (a) shows a near-linear increase in the short-circuit current density (Jsc) with increasing PTB7 thickness, rising from approximately 11.5 mA cm^−2^ at 100 nm to about 14 mA cm^−2^ at 1200 nm. This trend indicates enhanced optical absorption with increasing active layer thickness, particularly in the UV–visible region where PTB7 exhibits strong absorption due to its low bandgap (~1.81 eV). The absence of current saturation within the investigated range suggests that charge carrier extraction remains efficient up to 1200 nm, with recombination losses effectively mitigated under the assumed defect densities and mobility values.

Panel (b) presents the corresponding current density–voltage (J–V) characteristics for different PTB7 thicknesses. All curves exhibit nearly voltage-independent photocurrent over the applied bias range, with thicker PTB7 layers yielding proportionally higher current levels. This behavior indicates operation in a photoconductive regime, where photocurrent is governed primarily by carrier generation rather than injection barriers, and suggests that the internal electric field distribution is not significantly disrupted by increasing active layer thickness within this range.

In Panel (c), the external quantum efficiency (EQE) spectra are plotted as a function of wavelength for PTB7 thicknesses between 100 and 1200 nm. High EQE values are maintained across all thicknesses, particularly in the 320–450 nm ultraviolet–visible region, with peak efficiencies approaching ~80%. The weak dependence of EQE on thickness confirms that photocarrier collection remains efficient despite increased transport distance, while the gradual EQE decline beyond 600 nm reflects the intrinsic absorption cutoff of PTB7 in the near-infrared region.

Panel (d) shows the corresponding spectral responsivity curves, which peak between 400 and 450 nm, reaching values of approximately 0.43 A W^−1^ for the thickest PTB7 layers. As responsivity is directly proportional to EQE, this behavior further confirms efficient photoconversion in the targeted UV region. The suppression of responsivity at longer wavelengths contributes to intrinsic UV selectivity without the need for external optical filters.

Collectively, these results demonstrate that a PTB7 thickness of 1200 nm represents a practical upper limit, beyond which additional absorption gains are expected to be offset by increased carrier transit time and bulk recombination. The optimized thickness therefore maximizes UV absorption while maintaining efficient charge transport, leading to a high EQE, strong responsivity, and stable, bias-independent operation. These characteristics make the device particularly suitable for low-power and passive UV photodetection applications, including flame sensing, environmental monitoring, and biomedical diagnostics.

### 3.3. Effect of the Donor Density of the PTB7 Layer

The donor density in the PTB7 layer plays a critical role in governing the performance of organic photodetectors (OPDs) by directly influencing carrier transport, internal electric field strength, and recombination dynamics. PTB7 is a low-bandgap polymer with a high absorption coefficient and favorable charge transport properties, and its electronic behavior is therefore strongly coupled to controlled donor doping. In particular, donor density modulates the space-charge distribution within the active layer, thereby affecting band bending, exciton dissociation efficiency, and charge extraction.

**Figure 4:**
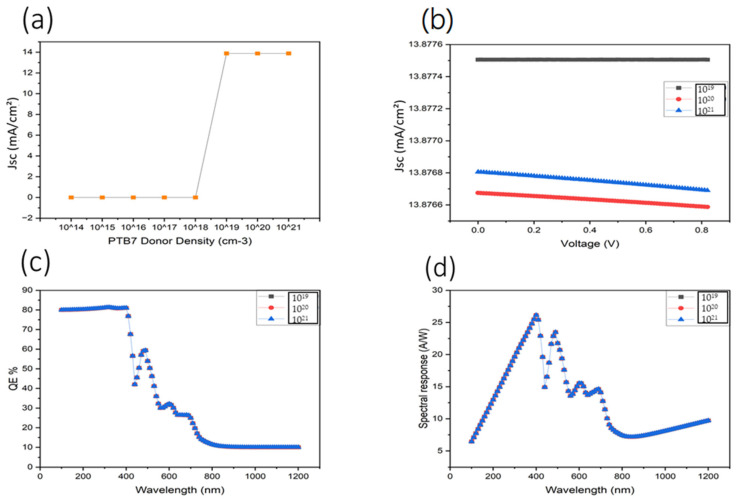
Impact of various PTB7 donor densities on device performance. (**a**) Donor density dependence of the current density. (**b**) Voltage–current density characteristics. (**c**) External quantum efficiency (EQE) as a function of wavelength. (**d**) Spectral response for different PTB7 donor densities.

The data presented in Figure 4 provide a quantitative illustration of the impact of PTB7 donor density on device performance. As shown in Panel (a), the short-circuit current density (Jsc) remains negligible for donor concentrations between 10^14^ and 10^18^ cm^−3^, indicating inefficient charge separation and extraction due to a weak built-in electric field. At these low doping levels, photogenerated carriers are dominated by recombination rather than field-assisted transport. In contrast, a sharp increase in Jsc to approximately 13.87 mA cm^−2^ is observed when the donor density reaches 10^19^ cm^−3^, marking a threshold at which sufficient space-charge density establishes a strong internal electric field. This enhanced field promotes efficient exciton dissociation and suppresses bulk recombination, enabling effective photocurrent generation.

Panel (b) shows the corresponding current density–voltage (J–V) characteristics for donor densities ranging from 10^19^ to 10^21^ cm^−3^. The overall shape of the curves remains stable, indicating robust device operation once the built-in field is established. A slight reduction in current density at donor densities beyond 10^19^ cm^−3^ suggests the onset of dopant-induced scattering and increased trap-assisted recombination, which can partially offset the benefits of stronger electric fields at excessive doping levels.

The external quantum efficiency (EQE) spectra shown in Panel (c) further support this interpretation. High and nearly identical EQE values of approximately 80% are achieved in the 320–400 nm ultraviolet region for donor densities ≥ 10^19^ cm^−3^, confirming that photoconversion efficiency is maximized once adequate internal field strength is reached. The minimal variation in EQE beyond this threshold indicates that carrier collection, rather than optical absorption, becomes the limiting factor. Minor oscillations at longer wavelengths are attributed to optical interference effects within the multilayer stack.

Panel (d) presents the corresponding spectral responsivity curves, which follow the same trend as EQE and peak near 400 nm with maximum responsivity values on the order of 0.4 A W^−1^ for optimized donor densities. No further enhancement in responsivity is observed at the highest doping levels, consistent with the J–V and EQE results and indicating that excessive donor density does not yield additional performance gains.

In summary, PTB7 donor density critically governs the balance between electric field-assisted charge extraction and recombination losses in OPDs. An optimal donor density of approximately 10^19^ cm^−3^ provides the best compromise, yielding high Jsc, strong EQE, and stable responsivity without introducing excessive recombination or transport limitations. These findings directly explain the abrupt Jsc transition observed in Figure 4 and highlight the central role of internal electric field formation in achieving high-performance, UV-selective organic photodetectors.

### 3.4. Effect of the Acceptor Density of the PTB7 Layer

The acceptor density in organic photodetectors (OPDs) can influence charge compensation, recombination dynamics, and carrier transport balance. However, its impact is generally weaker than that of donor doping in single-component or donor-dominated active layers such as PTB7, where charge transport and internal electric field formation are primarily governed by donor-induced space charge. Therefore, evaluating the sensitivity of device performance to acceptor density is important to identify parameters that can be safely de-emphasized during optimization.

**Figure 5:**
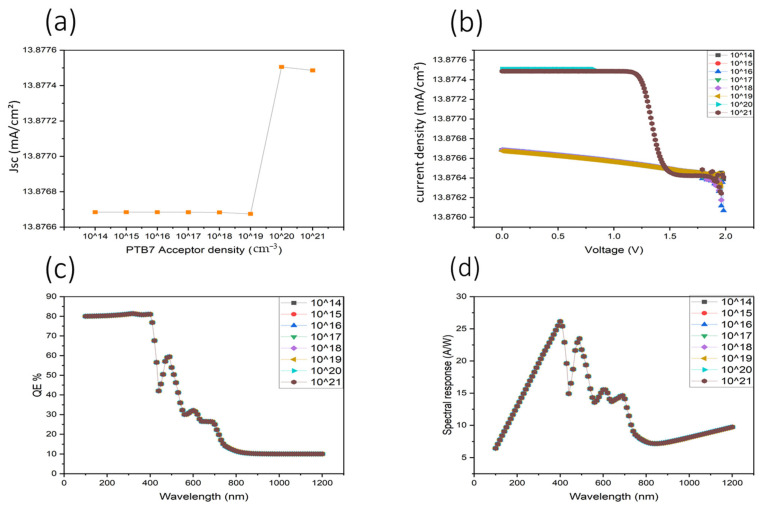
Influence of various PTB7 acceptor concentrations on device performance. (**a**) Current density versus PTB7 acceptor concentration. (**b**) Current density vs. voltage with varying acceptor density for PTB7. (**c**) External quantum efficiency (EQE)—wavelength with varying acceptor density for PTB7. (**d**) Spectral response (A/M)—wavelength (nm) for all acceptor densities.

The simulation results presented in Figure 5 provide a quantitative assessment of the influence of PTB7 acceptor density, varied from 10^14^ to 10^21^ cm^−3^, on device performance. As shown in Panel (a), the short-circuit current density (Jsc) remains nearly constant, varying only marginally from ~13.8766 to 13.8774 mA cm^−2^ across the entire acceptor density range. This negligible variation indicates that acceptor density does not significantly affect photocarrier generation or extraction in the present device architecture. Unlike donor doping, which directly enhances internal electric field strength and charge separation, acceptor doping plays a largely passive role in PTB7 under the simulated conditions.

Panel (b) further confirms this behavior, as the current density–voltage (J–V) characteristics are nearly identical for all tested acceptor concentrations. The overlapping curves indicate that charge transport, injection barriers, and recombination dynamics remain essentially unchanged, resulting in a stable photocurrent output under both forward and reverse bias regardless of acceptor density.

The external quantum efficiency (EQE) spectra shown in Panel (c) exhibit minimal dependence on acceptor concentration, maintaining peak values close to 80% in the 320–400 nm ultraviolet region. This spectral invariance confirms that exciton generation and dissociation are not limited by acceptor density in PTB7, and that photoconversion efficiency is dominated by intrinsic absorption and donor-side transport properties.

Similarly, the spectral responsivity curves in Panel (d) remain virtually unchanged across all acceptor densities, with consistent peak responsivity near 400 nm. The overlapping responsivity profiles further demonstrate that recombination and carrier extraction mechanisms are robust against variations in acceptor concentration within the explored parameter space.

In summary, the simulation results demonstrate that PTB7 acceptor density has only a marginal effect on OPD performance metrics, including Jsc, EQE, and responsivity. This behavior contrasts sharply with the strong sensitivity observed for donor density, highlighting an asymmetric dependence of device performance on doping type. Consequently, optimization of PTB7-based UV photodetectors should prioritize donor doping, active layer thickness, interface quality, and electrode work-function engineering, while acceptor density tuning can be considered a secondary parameter under standard operating conditions.

### 3.5. Spiro-OmeTad Donor Density

The donor density of Spiro-OMeTAD in organic photodetectors (OPDs), particularly in PTB7/Spiro-OMeTAD heterojunction systems, is a critical parameter that governs hole transport, interfacial band alignment, and recombination dynamics. As a widely used hole transport material (HTM), Spiro-OMeTAD offers high optical transparency, favorable energy-level alignment, and good film-forming properties. However, its electrical behavior is highly sensitive to donor doping, which alters space-charge distribution, band bending at the PTB7/Spiro-OMeTAD interface, and the internal electric field responsible for carrier extraction.

**Figure 6:**
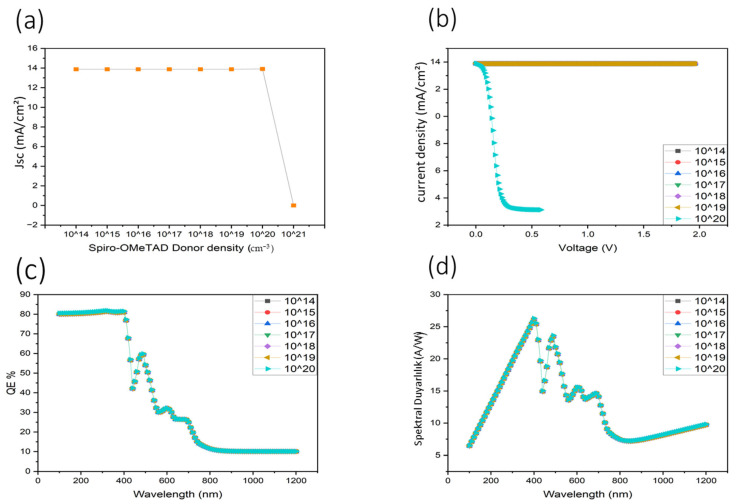
(**a**) Spiro-OMeTAD Donor Density (cm^−3^) versus Current Density, (**b**) J–V Characteristics, (**c**) Quantum Efficiency, (**d**) Spectral Response.

Figure 6 summarizes the influence of Spiro-OMeTAD donor density, varied from 10^14^ to 10^21^ cm^−3^, on key device performance metrics. As shown in Panel (a), the short-circuit current density (Jsc) remains nearly constant at approximately 13.87 mA cm^−2^ for donor densities between 10^14^ and 10^20^ cm^−3^, indicating stable hole transport and efficient extraction under these conditions. In this doping range, increased conductivity enhances transport without significantly perturbing the internal electric field. In contrast, at a donor density of 10^21^ cm^−3^, Jsc collapses abruptly to near zero, signaling a breakdown in charge collection.

This behavior is attributed to excessive space-charge accumulation within the Spiro-OMeTAD layer, which leads to over-bending of energy bands at the PTB7/Spiro-OMeTAD interface. Such over-doping can induce Fermi-level pinning and electrostatic screening of the built-in electric field, thereby suppressing field-assisted carrier separation and transport. As a result, photogenerated holes are no longer efficiently extracted, despite high HTL conductivity.

Panel (b) supports this interpretation, showing that the current density–voltage (J–V) characteristics remain stable and nearly voltage-independent for donor densities up to 10^20^ cm^−3^, while a complete collapse of photocurrent is observed at 10^21^ cm^−3^. The absence of measurable current at this extreme doping level explains why no meaningful J–V, EQE, or responsivity curves are observed for this condition, consistent with Reviewer 1’s observation.

The external quantum efficiency (EQE) spectra shown in Panel (c) further confirm these trends. For donor densities between 10^14^ and 10^20^ cm^−3^, EQE remains high at approximately 80% in the 300–450 nm UV region, indicating efficient photocarrier generation and collection. At 10^21^ cm^−3^, the EQE collapses across the entire spectrum, demonstrating that optical absorption no longer translates into extractable photocurrent due to severe transport disruption.

A similar trend is observed in Panel (d), where the spectral responsivity remains high and stable for donor densities up to 10^20^ cm^−3^, with a peak near 400 nm, but drops sharply at 10^21^ cm^−3^. This confirms that excessive donor doping in the HTL introduces parasitic electrostatic effects that dominate over conductivity gains, resulting in catastrophic device failure.

In summary, Spiro-OMeTAD donor density must be carefully optimized to balance enhanced hole conductivity with electrostatic stability. The simulations indicate that donor densities up to approximately 10^20^ cm^−3^ enable stable operation with high EQE and responsivity, while further increases lead to severe space-charge effects and interface disruption. These results highlight the critical role of doping engineering in hole transport layers and reinforce the necessity of controlled HTL doping for achieving high-performance, UV-sensitive organic photodetectors.

### 3.6. Effect of the Metal Electrode Work Function

Recent advances in organic photodetectors (OPDs) have highlighted the critical influence of the metal electrode work function on charge extraction efficiency and overall device performance. The work function of the back contact directly determines the energy-level alignment at the metal/HTL interface and therefore governs contact resistance, carrier injection barriers, and interfacial recombination. In particular, an improper alignment between the metal electrode and the highest occupied molecular orbital (HOMO) level of the hole transport layer (HTL) can lead to Schottky barrier formation, increased series resistance, and suppressed photocurrent. Conversely, a well-aligned high-work-function electrode facilitates near-ohmic hole extraction and minimizes contact-induced losses.

**Figure 7:**
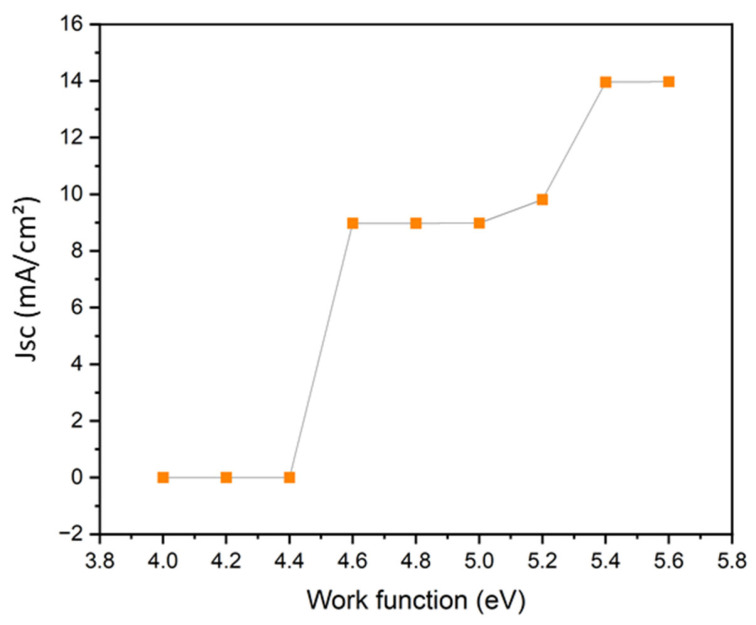
Variation in the current density with the metal work function.

In this study, the effect of varying the back-electrode work function from 4.0 to 5.6 eV was systematically investigated, as shown in Figure 7. When the work function lies in the range of 4.0–4.4 eV, the simulated current density remains nearly zero, indicating ineffective hole extraction due to the formation of a strong Schottky barrier at the electrode/Spiro-OMeTAD interface. This behavior is consistent with the large energetic mismatch between low-work-function metals and the HOMO level of Spiro-OMeTAD (~5.0 eV). As the work function increases from 4.5 to 5.2 eV, a rapid rise in photocurrent is observed, reaching approximately 9 mA cm^−2^, reflecting progressive reduction of the hole-injection barrier. This transition marks the onset of efficient field-assisted hole extraction as the electrode work function approaches the HTL HOMO level.

For work functions exceeding 5.2 eV, the current density increases further and eventually saturates near 14 mA cm^−2^ in the range of 5.3–5.6 eV, indicating the formation of a quasi-ohmic contact. In this regime, interfacial energy barriers are effectively eliminated, allowing photogenerated holes to be extracted without significant recombination or resistive losses. The plateau behavior suggests that, once ohmic conditions are achieved, further increases in work function yield diminishing returns.

Based on these results, gold (Au), with a work function of approximately 5.1–5.4 eV, was selected as the optimal back electrode in the final device architecture. Au provides excellent energetic alignment with the HOMO level of Spiro-OMeTAD, enabling efficient hole extraction, reduced contact resistance, and suppressed dark current. This choice also explains the inferior performance observed for Al-based contacts, which suffer from severe hole-injection barriers. In addition to its favorable electronic properties, Au offers high chemical stability, excellent electrical conductivity, and strong optical reflectivity, which collectively enhance both the electrical and optical performance of the photodetector.

In summary, the simulation results demonstrate that electrode work-function engineering is a decisive factor in optimizing OPD performance. The use of a high-work-function metal such as Au ensures proper energy-level matching at the HTL interface, leading to enhanced photocurrent generation, reduced interfacial losses, and improved overall device efficiency. These findings underscore the importance of contact optimization for achieving high-detectivity, UV-selective organic photodetectors.

### 3.7. Optimized Device and Responsivity

Following comprehensive simulations and systematic parameter optimization, the device architecture was refined to maximize photodetector performance, with a particular emphasis on ultraviolet (UV) operation. The optimal layer thicknesses were identified as 1200 nm for the PTB7 active layer and 1000 nm for the Spiro-OMeTAD hole transport layer, providing an effective balance between enhanced optical absorption and efficient charge transport. Parametric analysis of PTB7 donor density revealed that a concentration of approximately 1 × 10^19^ cm^−3^ yields optimal performance, as this doping level sufficiently strengthens the built-in electric field to promote exciton dissociation and charge separation without introducing excessive recombination losses. In contrast, variations in the PTB7 acceptor density were found to have a negligible effect on key performance metrics, confirming its secondary role in controlling device output within the investigated range. Similarly, that optimization of Spiro-OMeTAD donor density indicated that a value of 1 × 10^18^ cm^−3^ provides efficient hole transport while maintaining electrostatic stability at the PTB7/Spiro-OMeTAD interface.

The choice of back electrode was also critical to achieving optimal device performance. Gold (Au) was selected as the back-contact material due to its high work function (~5.3–5.4 eV), which closely aligns with the HOMO level of Spiro-OMeTAD and enables near-ohmic hole extraction. Compared with lower-work-function metals such as aluminum, Au significantly suppresses contact-induced recombination and dark current, thereby enhancing charge collection efficiency and device stability.

**Figure 8:**
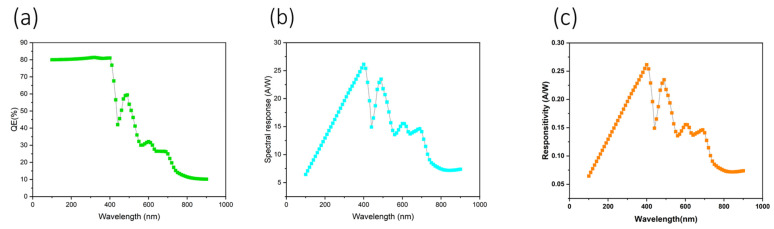
(**a**) EQE–wavelength relationship, (**b**) spectral response–wavelength, and (**c**) responsivity.

Figure 8 summarizes the optoelectronic performance of the optimized device. Panel (a) shows the external quantum efficiency (EQE) as a function of wavelength, exhibiting high values approaching ~80% in the ultraviolet region (300–400 nm), followed by a gradual decline at longer wavelengths consistent with the absorption characteristics of PTB7. Panel (b) presents the corresponding spectral responsivity, which peaks in the UV–blue region, confirming efficient photoconversion across the targeted UV spectrum. Panel (c) displays the responsivity curve, further validating the device’s ability to convert incident UV photons into measurable photocurrent with high efficiency and low background contribution from longer wavelengths.

Collectively, these results confirm the device’s strong specialization toward ultraviolet (UV) photodetection, as supported by both spectral and electrical performance metrics. Although the primary focus of this work is photodetector operation rather than power generation, the key parameters extracted from the simulated current–voltage (I–V) characteristics provide valuable insight into the charge extraction efficiency, contact quality, and overall electrical integrity of the optimized device. As summarized in Table 2, the device exhibits an open-circuit voltage (Voc) of 1.72 V, a short-circuit current density (Jsc) of 13.87 mA cm^−2^, and a high fill factor (FF) of 91.15%, resulting in a calculated power conversion efficiency (η) of 21.83% under simulated illumination. These values reflect efficient carrier separation, low series resistance, and minimal recombination losses rather than photovoltaic applicability. The maximum power point (VMPP = 1.60 V, JMPP = 13.60 mA cm^−2^) further indicates efficient charge extraction under operating conditions.

In summary, the optimized device architecture demonstrates highly promising characteristics for UV-selective organic photodetector applications. The combination of high EQE in the 300–400 nm ultraviolet range, suppressed long-wavelength response, low dark current enabled by optimized contacts and doping, and stable responsivity highlights the suitability of this design for applications such as flame detection, environmental monitoring, and biomedical sensing. While the present simulations target the near-UV region, the demonstrated optimization strategy provides a solid foundation for extending device operation toward narrower-band or solar-blind UV detection through future material and interface engineering.

To benchmark the performance of the optimized device, Table 2 compares the present work with representative organic and hybrid UV–visible photodetectors reported in the literature. Compared to previously reported devices, the PTB7/Spiro-OMeTAD photodetector investigated in this study exhibits competitive responsivity while operating at zero external bias, highlighting its suitability for low-power and self-powered UV detection. Notably, the optimized device demonstrates a strong photoresponse in the 300–400 nm ultraviolet range, which is achieved through intrinsic material absorption and contact engineering rather than a high reverse bias. These results confirm that the proposed architecture offers a favorable balance between responsivity, operating voltage, and spectral selectivity, underscoring the effectiveness of the simulation-guided optimization strategy.

## 4. Conclusions

This study presented a comprehensive, simulation-driven optimization of a high-performance organic photodetector (OPD) based on the PTB7/Spiro-OMeTAD material system, with a primary focus on ultraviolet (UV) photodetection. Through a systematic variation in structural and electronic parameters—including layer thicknesses, donor and acceptor densities, interface behavior, and the electrode work function—a physically consistent and optimized device architecture was identified that delivers strong photoresponse and stable electrical performance in the UV–visible range.

Key findings include the identification of optimal thicknesses of 1200 nm for PTB7 and 1000 nm for Spiro-OMeTAD, which maximize UV absorption while maintaining efficient charge transport. The optimal PTB7 donor density was determined to be approximately 1 × 10^19^ cm^−3^, providing sufficient internal electric field strength to enhance exciton dissociation and charge separation without introducing excessive recombination losses. In contrast, variations in PTB7 acceptor density exhibited only a marginal influence on device performance, highlighting the dominant role of donor-induced space charge in governing carrier dynamics. For the hole transport layer, a Spiro-OMeTAD donor density of 1 × 10^18^ cm^−3^ was found to yield optimal hole extraction while preserving electrostatic stability at the heterojunction interface. Furthermore, replacing aluminum with gold (Au) as the back electrode, owing to its higher work function (~5.3–5.4 eV), significantly improved energy-level alignment, resulting in enhanced hole extraction and reduced dark current.

The optimized device demonstrated strong electrical characteristics, including a short-circuit current density (Jsc) of 13.87 mA cm^−2^, an open-circuit voltage (Voc) of 1.72 V, and a high fill factor (FF) of 91.15%, reflecting efficient charge separation, low series resistance, and minimal recombination losses. Spectral analysis confirmed a high external quantum efficiency of approximately 80% and strong responsivity in the 300–400 nm ultraviolet region, validating the device’s suitability for UV-selective photodetection. The suppressed response at longer wavelengths indicates intrinsic spectral selectivity without the need for external optical filters.

Overall, this work underscores the importance of holistic optimization in OPD design and demonstrates that a careful control of layer thickness, doping profiles, interface quality, and electrode work-function engineering can enable highly sensitive, stable, and UV-selective organic photodetectors. While the present simulations focus on the near-UV region, the proposed optimization strategy provides a robust foundation for future experimental realization and for extending device operation toward narrower-band or solar-blind UV detection through targeted material and interface engineering. The findings offer practical design guidelines for next-generation organic photodetectors intended for environmental sensing, flame detection, and biomedical monitoring applications.

## Figures and Tables

**Table 1:** Physical parameters utilized for SCAPS-1D simulation.

Material Properties	FTO [36]	Spiro-OMeTAD [37]	PTB7 [38]
Thickness (nm)	500	100	50
Band gap (eV)	3.500	3.2	1.81
Electron affinity (eV)	4.000	2.1	3.31
Dielectric permittivity	9.000	3	3.4
Conduction band effective density of states, nc (cm^−3^)	2.20 × 10^18^	2.5 × 10^18^	1 × 10^20^
Conduction band effective density of states, nv (cm^−3^)	1.80 × 10^19^	1.8 × 10^19^	1 × 10^21^
Electron thermal velocity, Ve (cm/s)	1 × 10^7^	1 × 10^7^	1 × 10^7^
Hole thermal velocity, Vh (cm/s)	1 × 10^7^	1 × 10^7^	1 × 10^7^
Electron mobility, μe (cm^2^/Vs)	2.00 × 10^1^	2 × 10^4^	1 × 10^−3^
Hole mobility, μh (cm^2^/Vs)	1.00 × 10^7^	2 × 10^4^	1 × 10^−3^
Shallow uniform donor density, nD (cm^−3^)	2.00 × 10^19^	0	0
Shallow uniform acceptor density, nA (cm^−3^)	–	1 × 10^20^	0

**Table 2:** Comparison of responsivity and operating conditions of representative organic and hybrid photodetectors reported in the literature and the optimized PTB7/Spiro-OMeTAD UV photodetector developed in this work.

Photoactive Layer	λ [nm]	Bias [V]	R [mA/W]	Ref.
SnO2/CsPbI3/CuI	550–600	−0.5	400 @ 580 nm	[46]
PCDTBT and PC71 BM	400–700	−5	302 @ 500 nm	[47]
PTB7/Spiro-OMeTAD	300–400	0 (self-powered)	≈400 @ 400 nm	This work

## Data Availability

The data presented in this study are available on request from the corresponding author.
